# Determinants of individuals’ risks to 2009 pandemic influenza virus infection at household level amongst Djibouti city residents - A CoPanFlu cross-sectional study

**DOI:** 10.1186/1743-422X-11-13

**Published:** 2014-01-27

**Authors:** Fred Andayi, Pascal Crepey, Alexia Kieffer, Nicolas Salez, Ammar A Abdo, Fabrice Carrat, Antoine Flahault, Xavier de Lamballerie

**Affiliations:** 1 Aix Marseille Univ, IRD French Institute of Research for Development, EHESP French School of Public Health, UMR_D 190 "Emergence des Pathologies Virales”, 13005 Marseille, France; 2 IHU Méditerranée Infection, APHM Public Hospitals of Marseille, 13005 Marseille, France; 3 EHESP Rennes, Sorbonne Paris Cité, Paris, France; 4 Ministère de la Santé, Institut National de Santé Publique, BP 1157, Djibouti, Republic of Djibouti; 5 INSERM UMR-S 707, Université Pierre et Marie Curie Paris 6, Paris, France; Department of Public Health, Hôpital Saint-Antoine, AP-HP, Paris, France

## Abstract

**Background:**

Following the 2009 swine flu pandemic, a *cohort for pandemic influenza* (CoPanFlu) study was established in Djibouti, the Horn of Africa, to investigate its case prevalence and risk predictors’ at household level.

**Methods:**

From the four city administrative districts, 1,045 subjects from 324 households were included during a face-to-face encounter between 11th November 2010 and 15th February 2011. Socio-demographic details were collected and blood samples were analysed in haemagglutination inhibition (HI) assays. Risk assessments were performed in a generalised estimating equation model.

**Results:**

In this study, the indicator of positive infection status was set at an HI titre of ≥ 80, which was a relevant surrogate to the seroconversion criterion. All positive cases were considered to be either recent infections or past contact with an antigenically closely related virus in humans older than 65 years. An overall sero-prevalence of 29.1% and a geometrical mean titre (GMT) of 39.5% among the residents was observed. Youths, ≤ 25 years and the elderly, ≥65 years had the highest titres, with values of 35.9% and 29.5%, respectively. Significantly, risk was high amongst youths ≤ 25 years, (OR 1.5-2.2), residents of District 4(OR 2.9), students (OR 1.4) and individuals living near to river banks (OR 2.5). Belonging to a large household (OR 0.6), being employed (OR 0.5) and working in open space-outdoor (OR 0.4) were significantly protective. Only 1.4% of the cohort had vaccination against the pandemic virus and none were immunised against seasonal influenza.

**Conclusion:**

Despite the limited number of incident cases detected by the surveillance system, A(H1N1)pdm09 virus circulated broadly in Djibouti in 2010 and 2011. Age-group distribution of cases was similar to what has been reported elsewhere, with youths at the greatest risk of infection. Future respiratory infection control should therefore be tailored to reach specific and vulnerable individuals such as students and those working in groups indoors. It is concluded that the lack of robust data provided by surveillance systems in southern countries could be responsible for the underestimation of the epidemiological burden, although the main characteristics are essentially similar to what has been observed in developed countries.

## Background

In April 2009, an acute febrile respiratory illness that spread rapidly across Mexico and the United States [1], was reported. This aetiological pathogenic virus was later identified as a new influenza A strain (referred to as A(*H1N1)pdm09 virus* in this article), a re-assorted variant of North American and Eurasian swine lineages which was immunologically distinct from the circulating seasonal influenza A strain H1N1s [2]. The geographic dispersion of this virus resulted in high numbers of new cases that overwhelmed laboratories and the clinical capacity of many nations, compelling the WHO to issue a pandemic alert on June, 11th 2009 [1]. A year later, more than one million cases and almost 20 thousands deaths had been reported from 214 countries [3]. These figures are likely to be an underestimate of the actual morbidity and mortality burden due to the *A(H1N1)pdm09 virus*, particularly amongst southern hemisphere nations [4]. The WHO further encouraged the scientific community to investigate the severity of this new pathogen and the associated risk factors. Amongst the notable observations were the high antibody titres mainly thought to be due to new infections amongst the young population (≤ 25 years) and previous contact with the antigenically related H1N1 strain amongst the elderly (above 65 years) [5,6]. More severe cases and fatalities were observed in young people, co-morbidity conditions [7], obese and pregnant women [6,7]. At that time, available data on Influenza burden estimation were mainly derived from North hemisphere countries, plus Australia and New Zealand but were severely lacking in many other southern countries. Disparities in the influenza funding programme, healthcare systems and research activities, were the other important significant contributory factors [8]. The southern group also contained a high prevalence of other infectious agents such as HIV, malaria, Tuberculosis, malnutrition and hygiene related gastroenteritis [9]. Under the WHO region classification, African and Eastern Mediterranean (WHO-EMRO) countries, have a high prevelance of these pathogens which has not been systematically documented. Djibouti, the country of interest in this study, is one of 22 member states belonging to the WHO-EMRO region. The other countries in the region include, *Afghanistan, Bahrain, Djibouti, Egypt, Iran, Iraq, Jordan, Kuwait, Lebanon, Libya, Morocco, Oman, Pakistan, Qatar, Saudi Arabia, Somalia, Sudan, Syria, Tunisia, United Arab Emirates* and *Yemen.* In Djibouti, the ILI (influenza like illnesses) laboratory confirmed surveillance data are a work in progress and therefore syndromic reporting represents the backbone of disease monitoring. According to the recent annual health statistics report [10], influenza and ILI account for most of the consultations and incidence cases in health facilities, in particular accounting for 48% and 53% of all clinical consultations reported among adults and children. In the same category, 47% and 46% of all incidence cases reported, respectively [10]. These data therefore underscore the need for sound monitoring of ILI by identifying the aetiological agents and their associated risks, to allow early detection and advance preparedness against serious outbreaks such as the recent swine flu pandemic. Here, we have focussed on Djibouti, a subtropical country faced with the previously cited challenges. We report sero-prevalence data from a cohort of 1,045 subjects and uncover risk determinants of A(H1N1)pdm09 infection amongst Djibouti city residents during the A(H1N1)pdm09 pandemic.

## Method

The Djibouti CoPanFlu *(Cohorts for Pandemic Influenza)* programme was part of an international project that performed sero-epidemiological investigations of influenza A(H1N1)pdm09 that was identified in six countries from five different continents (France [11,12], Laos [13], Djibouti [14], Mali [15], Bolivia (Delangue et al., manuscript in preparation), and the Indian Ocean (Reunion Island [16]) through longitudinal prospective household cohort studies. All centres used a standardised sampling and testing protocol, three phases and two years follow up, adapted to the local context of the host country [11]. Unlike other centres, Djibouti’s study was limited to Phase one, and therefore is hereby reported as a cross-sectional study.

### Study design and demographic characteristics

**Figure 1:**
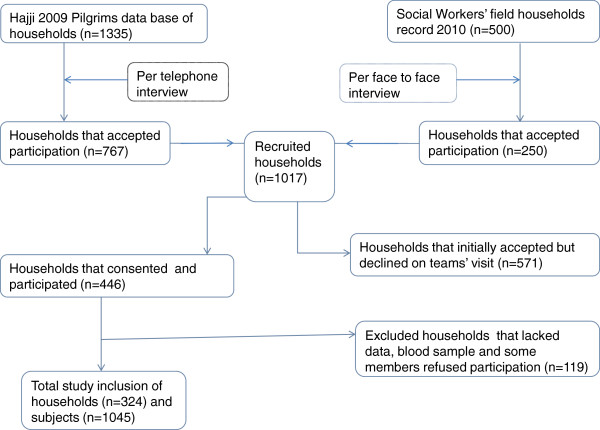
Inclusion chart showing the various stages included during the constitution of the 324 households and 1,045 subjects from amongst the residents of Djibouti city.

The study was conducted between 11th November 2010 to 15th February 2011 in four administrative districts (*arrondissements*) of Djibouti city, which is the largest urban agglomeration and capital city of the Republic of Djibouti, a country in the Horn of Africa. It covers about 23,200 km^2^ and hosts 818,159 inhabitants, the majority of whom 58.1% (475,322) are inhabitants of Djibouti city [17]. It has two climatic seasons, the summer which lasts from May to September, and the winter from November to April [18]. After receiving authorisation from relevant government departments, 1,835 household heads were recruited from two sources: 1,335 were from the 2009 Hajj Pilgrim database and 500 from the community of health workers (CHW) cognisance list (Figure 1). The Hajj Database is an annual document constituted by the Djibouti Ministry of Religious Affairs and Immigration for participants to Muslim pilgrimage to Mecca, Saudi Arabia. The CHW database is a document constituted by the Djibouti Ministry of Health. It includes a list of vulnerable households earmarked for emergency government support in case of natural disaster or disease outbreaks. Information was given to all household members and enrolment was conducted when all members could be included. Participants or their legal representatives were *a priori* required to give informed consent. Only households meeting the following criteria were enrolled in our cohort; all members of the household shared one roof, they shared meals and living area, consented to participation (including blood sampling and responding to questionnaires), and were permanent residents of District 4. On an appointed date, the capillary blood samples (~100-500 μL) were collected and the assisted response to standardized French questionnaires was completed, using the local dialect to translate questionnaires whenever necessary. The fresh blood was allowed to clot at room temperature, before separating sera from clots by centrifugation. Separated sera were then stored at -20°C until the assay time. All assays were conducted in Biosafety level 3 laboratory environments at the EPV UMR_D 190 laboratory of the University of Aix Marseille 13005, France.

### Laboratory analysis

Detection of antibodies to A(H1N1)pdm09 virus was performed according to CoPanFlu standardized HI protocols, as previously reported [13,14,19]. This entailed twofold automated dilution 10^-1^ to 10^-7^ of test samples and control (positive and negative) sera, performed in the presence of a serum non-specific agglutination inhibitor. A highly specific cut off of HI titre at ≥80 was used to identify positive samples.

For the detection of sero-neutralisation antibodies, we performed analysis on the HI positives (≥80) using a standard microneutralisation (VNT) assay protocol [12]. It entailed an automated twofold serial dilution 10^-1^ to 10^-7^of test samples and control sera in flat bottomed 96-well cell culture microplates (Nunc™). A 50 μL sample of titrated virus at 100TCID_50_ was then added to an equal volume of serum and incubated at 37°C in a CO2 incubator for 60 minutes. Afterwards, a 50 μL aliquot of freshly prepared MDCK cell culture suspension at 2 × 10^5^ cells/μl was added, and then incubated at 37°C in a CO2 incubator until the cytopathic effect (CPE) formed in the control, which was usually about 3-5 days. Absence of CPE was considered to reflect complete neutralisation (positive reaction). A serum with standard VNT titre at ≥10 was considered to be positive [20,21].

### Data management and analysis

The wealth index (SES class) was determined on the basis of household ownership of nineteen different assets, by the *principal component analysis* as described by Vyas et al [22]. The GMT computation was conducted according to the SAS PROC LIFEREG, which is a survival analysis procedure in SAS 9.3 statistical software. The method avoids underestimation of the censored observations in the calculation of GMT as described by Nauta [23]. In brief, a titre of 5 was assigned to all HI tests resulting in negative observations, followed by a log transformation of log HI titre = log2 (HI titre/5), and estimation of the maximum likelihood of the GMT of truncated HI titres and their 95% confidence interval. To avoid potential bias, all the vaccinated subjects were excluded from all the prevalence and risk analyses. The risk analysis was performed in the generalised estimating equation model to determine the predictors of individuals’ infection at household level. This model accounts for the existing correlation between subjects enrolled from the same household. Infection status (HI titre ≥ 80) was the dependent variable that was evaluated against several independent variables from socio-demographic, housing environment and subject profile. Those variables found to have p-value <0.25 in bivariate analysis, were fitted in a multivariate model through a backward stepwise reduction process in accordance with the Bursac et al [24] method. In the final model, variables with p-value ≤ 0.05 were considered to be statistically significant.

### Ethical statement

Ethical approval was received from the WHO-EHESP CoPanFlu International Consortium of the *French School of Public Health (EHESP)* in France [11] and the Ethical Review Committee of the Djiboutian Ministry of Health’s *National Institute of Public Health (INSP)*. A written informed consent was obtained from each study participant, including the minors (<16 years) through their parents or guardians.

## Results

### Demographic characteristics

From the four administrative districts of the city, 1,045 subjects, belonging to 324 households, were included in the CoPanFlu cohort (Table 1 and Figure 1). The overall median age was 25.9 (range 0.3-100.9) years, and there were no significant differences in age distribution between sample and the national population census [17]. We had an average of 3 (range 1–10) members per household who were stratified into three groups: 563 subjects (53.9%) were living in *“large households”* (≥5 persons), 152 (14.6%) in *“medium households’* (3–4 persons) and 330 (31.6%) in *“small households”* (1–2 persons)*.* Within these households, 72% (755) of the subjects belonged to a household with children. Subjects were distributed according to occupation, there were four different categories: (a) “jobless” (45.3% (473)) were defined as persons above 13 years, not in any gainful economic activities, including the retirees and those who were not attending school/university; (b) “students” (28.8% (301)) were persons attending school/university most of the day, no age limit; (c) the “employed” (15.5% (162)) were persons in gainful economic activities, public or private, as a means of livelihood; (d) “under 13 years and not student” (10.4% (109)) were persons below 13 years of age, who were neither employed nor schooling. Concerning literacy levels, 25.4% (265) had no formal training, 19.2% (201) had basic education, 11.7% (123) had high-school education; missing values: 43.7% (456). On wealth index (SES), three classes were computed, 27.3% (285) belonged to the lowest class, 12.2% (127) to the middle class and 37.5% (392) to the upper class; missing values: 23% (240). Of the constituted subject cohort, 1.4% (14) had been vaccinated against A(H1N1)pdm09 and none for seasonal influenza, 8.1% (85) had on-going chronic illness, 10.5% (110) had a recent respiratory infection (ILI) illness in the past three months from November 2010), 1.3% (14) were on an unspecified treatment and 2.3% (13) of the women were pregnant.

**Table 1:** Demographic characteristics of the Djibouti CoPanFlu cohort

***Demographic characteristics***	***Number of individuals in the cohort***	***Proportion in the cohort***
**Gender (female)**	571	54.64%
**Age**		
0-14 year-old (yo)	332	31.77%
15-24 yo	166	15.89%
25-54 yo	472	45.17%
55-64 yo	40	3.83%
>65 yo	25	2.39%
Unknown	10	0.96%
**Localisation**		
District 1	433	41.44%
District 2	340	32.54%
District 3	200	19.14%
District 4	72	6.89%
**Ethnic origin**		
Afars	101	*14.64%*
Arabs	174	17.50%
Somalis	645	64.90%
Ethiopians	58	5.80%
Migrants	15	1.50%
Unknown	52	4.98%

**Figure 2:**
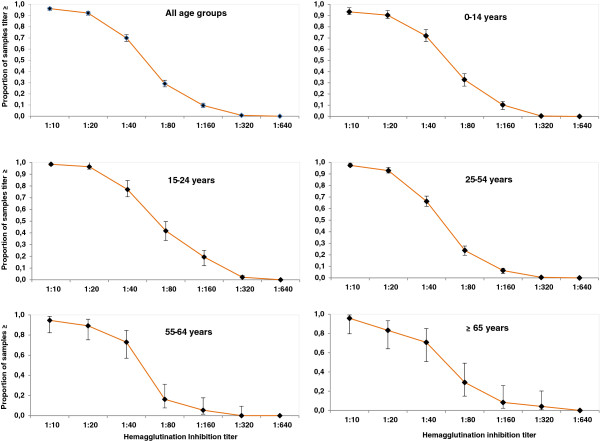
Reverse cumulative distribution curves of HI titres for antibodies and their 95% confidence intervals against H1N1pdm09 for different age groups amongst Djibouti city residents (Nov 2010-Feb 2011).

### Influenza serological status in age groups

**Figure 3:**
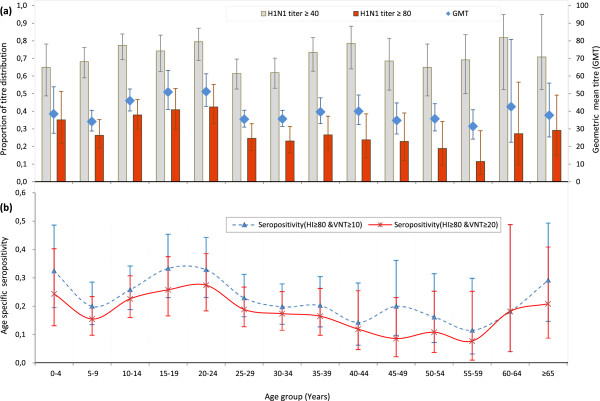
**Relationship between Djiboutian age group and level of antibodies against H1N1pdm09 virus.** The graphs illustrate the relationship between age groups to; **(a)** the specific antibodies (titres ≥40, ≥80 and GMT) and to that of **(b)** the neutralising antibodies (titres ≥10 and ≥20), with their 95% confidence interval.

In this study, the infection status (seropositivity) was determined by our established HI assay protocol [13,16,19,25], in which, the HI titre at ≥ 80 is considered a cut off when the use of seroconversion criteria is impractical, since the two measures have been confirmed to approximate each other [13,16,19,25]. All positive cases were attributed to the recent infection or past contact with an antigenically closely related virus in those older than 65 years [5,6]. Of the 110 subjects with recent respiratory infections, 23 were seropositive for the 2009 pandemic influenza virus. Figure 2 shows the reverse cumulative distribution of HI titres for antibodies against A(H1N1)pdm09 by various age group categories. The relationship between age group and level of antibodies is further detailed in Figure 3, which reports (a) seroprevalence values in age classes according to ≥40 and ≥80 HI titre cut-off values, and HI GMT values and (b) distribution of neutralising antibodies seropositivity in subjects with HI titre ≥80 (according to ≥10 and ≥20 VNT titre cut-off values). The main observations are: **(i)** The poor discrimination in age groups when using an HI cut-off at 40, is in agreement with previous studies using the same experimental protocol [13]. HI results at titre 40 presumably aggregate the detection of low-titre specific antibodies and cross-reactive antibodies previously acquired following seasonal influenza infection [12]. The HI cut-off at ≥80 is more discriminative and provides distribution in age groups that faithfully follows that of neutralising antibodies (Figure 3). **(ii)** The global “U-shaped” distribution of antibodies in age groups, with a lower prevalence of antibodies in subjects belonging to the 25–60 age classes. Both the prevalence at HI ≥ 80 and the mean HI titre were significantly lower in this group, when compared to youths below 25years. As discussed elsewhere, this distribution may reflect, on the one hand the epidemiological exposure of the children and young adults to influenza infection, and on the other hand past exposure to H1N1 variants (that share more antigenic similarity with the novel pandemic variant than recent seasonal H1N1 strains) for patients over the age of 60 (*i.e.* ‘pre-drift’ Spanish flu-related H1N1 strains) [2,5].

### Seroprevalence according to districts of Djibouti city

**Figure 4:**
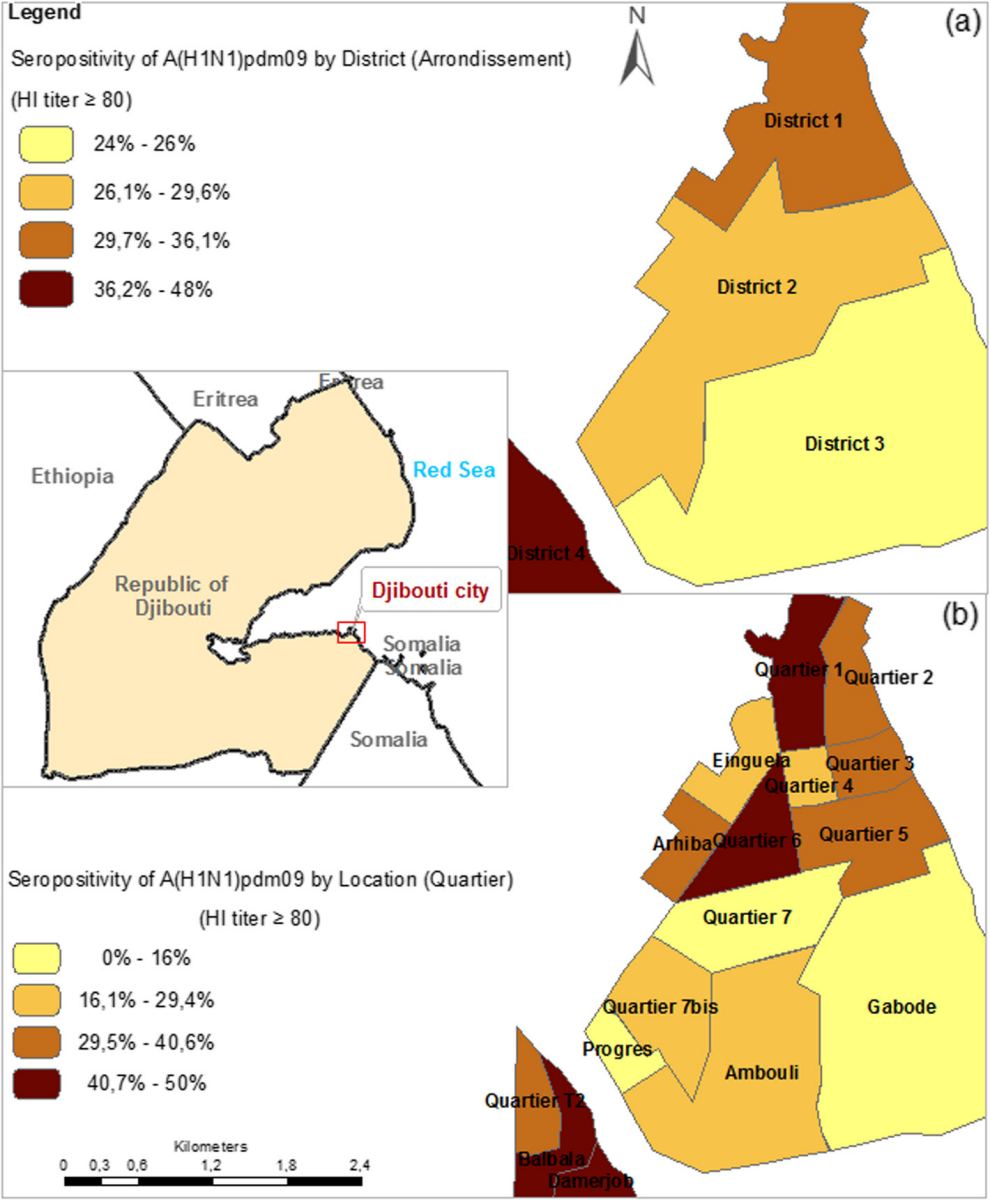
Map showing the geographical location of the study area, the Djibouti city within the republic of Djibouti and the prevalence of A(H1N1)pdm09 in administrative Districts (a) and locations (b) and as determined by a titre of ≥80.

In a choroplethic map, seroprevalence of antibodies was highest in *District 4,* followed by *District 1, District 2* and *District 3,* in that order (Figure 4a). A refined analysis according to Locations “Quartiers” identified Quartiers 1, 6, Balbala and Damerjob as those with the highest observed seroprevalence values (≥40.7%) (Figure 4b).

### Regression analyses

From the bivariate analysis, significant associations were confirmed between infection status and a number of risk factors, namely; *age group, place of residence, occupation, working environment, household size* and *living nearby river bank (*ie habitants living near River Balbala valley banks, drawn from *District 2, District 3* and *District 4*.); but not for others, such as, *recent respiratory illness, wealth index (SES), gender, having children in the household, literacy level, pregnancy in females, tribe, chronic illness* and *other housing environment variables* (Table 2). In the final multivariate model, *working environment*, *occupation* and *wealth index (SES)* were significant risk predictors of A(H1N1)pdm09 infection*.* With the exception of *occupation-student* (aOR 2.2, p = 0.0075), the two others, working *in open air space* (aOR 0.4, p = 0.0253) and belonging to low SES class (aOR 0.4, p = 0.0348), were protective against the risk of infection (Table 2).

**Table 2:** List of predictors of influenza H1N1pdm09 infection amongst Djibouti city residents

	**Population characteristics**	**Univariate analysis (OR-odd ratio)**			**Multivariate analysis (adjusted aOR-odd ratio)**		
		**OR 95% CI**	**Std error**	**P-value**	**OR 95% CI**	**Std error**	**P-value**
**1**	Age group (0-14 yrs)	1.5 (1.1-2.2)	1,18	**0.0105**	0.8 (0.1-6.1)	2,80	0,8413
	15-24 yrs	2.2 (1.5-3.3)	1,23	**<.0001**	1.2 (0.5-2.9)	1,55	0.6189
	25-54 yrs	Ref	Ref	Ref	Ref	Ref	Ref
	55-64 yrs	0.3 (0.3-1.5)	1,58	0.3002	0.4 (0.4-4.0)	3,26	0,4256
	>65 yrs	1.3 (0.5-3.3)	1,58	0.5339	-	-	-
**2**	District (1)	1.3 (0.8-2.0)	1,26	0.2500	2.0 (0.5-5.5)	1,67	0,1822
	2	1.2 (0.8-1.9)	1,26	0.3453	1.7 (0.7-4.0)	1,57	0,2668
	3	Ref	Ref	Ref	Ref	Ref	Ref
	4	**2.9 (1.4-5.8)**	1,42	**0.0027**	1.9 (0.7-5.5)	1,72	0.2358
**3**	SES class(Low)	0.6 (0.4-1.1)	1,32	0.1142	0.4 (0.2-0.9)	1,53	**0.0348**
	Upper	0.7 (0.4-1.2)	1,31	0.2347	-	-	-
	Middle	Ref	Ref	Ref	Ref	Ref	Ref
**4**	Household size: Large (≥5 persons)	**0.6 (0.4-0.9)**	1.20	**0.0105**	-	-	-
	Medium (3–4 persons)	0.8 (0.5-1.3)	1.30	0.3102	-	-	-
	Small (≤2 persons)	Ref	Ref	Ref	Ref	Ref	Ref
**5**	Occupation (<13 yrs and not student)	0.9 (0.5-1.5)	1,34	0.6157	-	-	-
	Employed	0.5 (0.3-0.8)	1,27	**0.0067**	-	-	-
	Students	**1.4 (1.0-2.0)**	1,18	**0.0313**	2.2 (1.2-3.9)	1,34	**0.0075**
	Jobless	Ref	Ref	Ref	Ref	Ref	Ref
**6**	Gender (Man)	0.9 (0.7-1.2)	1,16	0.5588	-	-	-
**7**	Have waste bin	0.7 (0.5-1.1)	1,23	0.1570	-	-	-
**8**	Live nearby large dumpsite	0.7 (0.4-1.1)	1,28	0.1264	-	-	-
**9**	Live nearby industry	4.8 (0.5-41.3)	2,99	0.1521	-	-	-
**10**	Live nearby river banks	**2.5 (1.2-5.1)**	1,44	**0.0119**	-	-	-
**11**	Live nearby small dumpsite	0.6 (0.4-1.1)	1,30	0.0883	-	-	-
**12**	Khat use	0.7 (0.5-1.1)	1,22	0.1381	-	-	-
**13**	Smoking	0.7 (0.5-1.0)	1,23	0.0826	-	-	-
**14**	Chronic illness	0.9 (0.5-1.6)	1,33	0.6938	-	-	-
**15**	Recent Respiratory illness	1.0 (0.6-1.7)	1,30	0.9554	-	-	-
**16**	Have children in household	1.2 (0.8-1.7)	1,20	0.3973	-	-	-
**17**	At night may sleep out, in the open	1.4 (0.8-2.6)	1,34	0.2154	-	-	-
**18**	Working in indoor space area	0.6 (0.3-1.1)	1,35	0.1132	-	-	-
**19**	Working in open space area	**0.4 (0.2-0.8)**	1,43	**0.0105**	0.4 (0.2-0.9)	1,44	**0.0253**

Estimation used generalised estimating equations. Predictors in univariate analysis with p-value ≤0.25 were included in the multivariate analysis. Seropositivity was based on an HI titre cut-off of ≥1:80. Predictors with p-value >0.05 were considered significant and are presented in bold.

## Discussion

Our results revealed that: the younger people were amongst the most affected; that certain regions of the city bore a disproportionately high risk for a pandemic burden compared with other regions and that the occupation and working environment of individuals were important A(H1N1)pdm09 infection risk predictors for Djibouti. We therefore report for the first time, the epidemiological characteristics of pandemic influenza A(H1N1)pdm09 in this nation, which is located in the WHO Eastern Mediterranean Region (EMR). EMR is comprised of 22 countries, including Djibouti (see introduction) and has little available epidemiological information for A(H1N1)pdm09, particularly relating to morbidity (incidence), mortality (fatalities) and sero-prevalence (sero-surveillance). The EMR is in the northern hemisphere. According to recent studies, the global pandemic occurred in two major waves in both the southern and northern hemispheres [4]. In many parts of the world, the first wave occurred between May and November 2009, followed by a gap and overlap by an ascending second wave that ran from October 2009 to February 2010 [4,26]. Once introduced, the A(H1N1)pdm09 virus spread rapidly across the country for 15 to 20 weeks, thereafter transmission was sustained at a much lower intensity [27]. This strain had the ability to predominate and replace other seasonal strains like the H3N2 and seasonal H1N1, with few exceptions.

As documented in temperate countries [27], in EMR the two waves seem to have followed and peaked in cold times during winter, but their peak magnitude varied between countries. The first pandemic wave is presumed to have begun with the reports of the first cases in the region, which were in Kuwait in May 2009 and in the United Arab Emirates (UAE) in July 2009 [28,29], before spreading to other member states. Once infection was introduced to these two nations it gradually progressed to allow community transmission, peaking in August 2009 for UAE, and in October 2009 for Kuwait [28,29]. The second wave was more pronounced and better documented than the first, probably due to more awareness of the infection. It begun in late October 2009, peaked in December 2009 and January 2010 and then declined from February to March 2010 [3], with a lag in Afghanistan, Iraq and Oman [3,26]. This corroborates the sentinel data in Egypt and Morocco [30], which confirmed that the peak of A(H1N1)pdm09 incidence occurred between November 2009 and January 2010. Similarly, to Djibouti’s neighbour, Ethiopia, which reported its first two cases in June 2009 (first wave), followed by a lapse until early 2010 (second wave), when more cases were noticed [31]. Although this scenario was acceptable to the regional WHO EMR office, it should be considered carefully since countries with functional surveillance systems were likely to detect and report the pandemic circulation earlier than those without these surveillance systems.

The EMRs official statistics of 24th October 2009, indicated 17,150 incidences and 111 fatalities [32]; and later, as of 30th April 2010, reported 1,059 fatality cases [26]. Amongst the 1,059 fatalities reported, Egypt (277), Iran (147), Saudi Arabia (128), Syria (138) and Morocco (64) had the highest proportion in 20 of the 22 member states involved [26]. At the country level, limited Information was available from Djibouti, with only nine laboratory confirmed cases and zero deaths cited in both reports. Unpublished influenza and influenza like illness data suggest that Djibouti city might have experienced a lagged second wave with a steady increase from January to June and a sharp decrease in July (Dr. Ammar Abdo Ahmed, personal communication). This is, however, based on a quite limited number of cases (n = 278). We therefore observed that it was difficult to propose an estimate of the actual epidemic impact, because the different patterns reported from neighbouring countries (*e.g.*, Ethiopia [31]) indicated that incident cases might have been underreported. We attempted to provide additional epidemiological information through this seroprevalence study of the Djiboutian population, which happened in the winter of 2010 (November 2010 to February 2011).

This study had several limitations. Firstly, late sampling did not enable distinction between individuals infected during the very first wave in summer 2009 and those infected during the second wave (autumn 2009 and during 2010). The general assumption was that the infection occurred between the winter of 2009 (November 2009 to February 2010) and that of 2010 (November 2010 to February 2011), with the exception of subjects over 65 years old who could have been exposed to pandemic antigenically related strains before [5,6]. As a result, the study could only provide a rough cumulative estimate of the influenza A(H1N1)pdm09 burden during years 2009–2010. Secondly, our study potentially underestimated the prevalence of pandemic influenza since the cohort data collection ended one month before the end of the winter season. However this underestimation was unlikely to have any significant effect on epidemiological characteristics such as age, spatial distribution and risk factors. Thirdly, in the absence of pre-pandemic samples, sero-prevalence data were used as a surrogate for seronconversion data to estimate the number of infected cases. Of note is that this approach has been validated to be a robust estimation of seroconversion rate as is evident from previous studies [15,16]. Fourthly, the opportunistic study of individuals recruited from the 2009 Hajj Pilgrim and community health (social) workers databases cannot claim to provide a representative picture of the Djiboutian population. Hypothetically, we might have compromised the participation of the middle class households since the Hajj Pilgrimage to Mecca is costly and only those amongst the high-income bracket can afford to do it, and, conversely, it is the majority of low-income persons who depends on the community health (social) workers service. These two groups are therefore likely to have different exposures to infection; and international travel and participation in the Hajj could have elevated the risk to pandemic influenza to those who were involved [33]. However, with regard to influenza epidemiology, such a bias was expected to have a limited impact on the estimate of the global number of cases in the general population [34].

In our study, which happened soon after the second pandemic wave (November 2010- February 2011), we had a 29.1% overall seroprevalence and with most occurring amongst the young, 35.1% in 0–4 years old and 34.5% in 5–19 years old. These observations are consistent with the recent meta-analysis estimation of ten studies from nine countries (of Asia, Africa, Europe and North America) [4], which indicate that the overall prevalence, in the same period, was 32% (95% CI 26-39%) and more occurrences were in 5–19 year olds at 47% and in 0–4 year olds at 36%, as was the case of Djibouti [4]. We also compared the prevalence (34.4%) amongst young Djiboutian <20 years with two other countries, Laos [13] (20.8%) and Mali [15] (19.5%) that applied the study protocol. A brief overview of the EMRs status, based on the first 500 reported cases, indicates more young people (29 years) were disproportionately affected in the first wave and accounting for up to 59.6% of all incidences [26]. However, precise comparison with findings from other regions is hampered by the limited information reported [26], and variations in the occurrence of the first and second waves [35,36]. Our findings suggest that the country had a comparable higher disease burden than many of the other documented countries. This therefore usefully complements data from the Djibouti Ministry of health, the regional health agencies, the WHO and the CDC records.

In the risk assessment for A(H1N1)pdm09 infection, our results indicated that the seropositivity was significantly associated with individuals’ age (<25 years), occupation (student), place of residence (District 4) and the living environment (living near to a riverbank). As mentioned earlier, people under 25 years old were immunologically naïve and vulnerable to infection with the new virus [5,6]. Amongst the elderly, cross-reactive antibodies against the 2009 pandemic influenza virus are likely to have originated from infections caused by the 1918 Spanish influenza virus and antigenically related descendants as suggested by haemagglutinin sequence comparison [6]. Similarly, students may be at specific risk of infection because of their age class and as a result of their close social interactions within the school environment that potentiates rapid spread of ILI [6]. By residential location, we observed that District 4 had the highest risk for infection compared to those residing elsewhere. This region is geographically separated from the three others, districts 1, 2 and 3, by a seasonal river. This population is largely made up of refugees or illegal migrants fleeing the civil unrest in neighbouring countries, living in close proximity and have high material deprivation. Such characteristics in part contributed to their underrepresentation (and that of the district) in our cohort, due to the very restrictive household enrollment protocol applied. In spite of this, the overall picture of seropositivity distribution by age group and risk factors was unlikely to be biased. Because, the age group proportions of the district was not significantly different from those of the three other districts. Whilst low wealth index alone was not associated with seropositivity (see below), it is possible that specific characteristics of this vulnerable population may represent an increased risk of infection [34]. Besides District 4, individuals living close to the River Balbala valley banks (see Figure 4, comprised of some residents from *District 2, District 3* and *District 4)*, had a three times greater risk of infection than those living further away. We have no specific explanation for this observation.

Of note is the observation of significant protection against the A(H1N1)pdm09 infection by those people having a low wealth index, large household size, being working class and working in open air spaces. A possible explanation is that the Djiboutian people belonging to a low wealth index class could have had limited exposure to the new pandemic strain, hence the lower incidence [34], a situation mostly associated with relative social isolation compared to other groups such as the working class who would benefit from better living standards, access to medical treatment and information. Similarly, people working in areas which are well ventilated would have limited chance of sustained air contamination from infectious aerosols. According to Kieffer *et al.*, the lower risk amongst households with more members was attributed to the overcrowding, which results in reduced times that one member spends face-to-face with another, thereby decreasing exposure times between individuals, and thus limiting the infection risk [13].

## Conclusion

Despite the limited number of incident cases detected by the surveillance system, A(H1N1)pdm09 virus circulated broadly in Djibouti in 2010 and 2011. Age-group distribution of cases was similar to what has been reported elsewhere, with youths at the greatest risk of infection. Future respiratory illness control should therefore be tailored to reach specific and vulnerable individuals such as students and those working in groups indoors. It is concluded that the lack of robust data provided by surveillance systems in southern countries could be responsible for the underestimation of the epidemiological burden, although the main characteristics are essentially similar to what has been observed in developed countries.

## Competing interests

The authors declare that they have no competing interests.

## Authors’ contributions

AF: field work, lab analysis, statistical analysis, and first draft manuscript writing; CP: statistical analysis, and manuscript writing; KA: field coordination, data management; SN: lab analysis; AAA: field coordination; FA: study design; and DLX: study design, and coordination. All authors read and approved the final draft.
